# The Effects of Three Remineralizing Agents on the Microhardness and Chemical Composition of Demineralized Enamel

**DOI:** 10.3390/ma14206051

**Published:** 2021-10-13

**Authors:** Ivan Salinovic, Zdravko Schauperl, Marijan Marcius, Ivana Miletic

**Affiliations:** 1 Department of Endodontics and Restorative Dentistry, School of Dental Medicine, University of Zagreb, 10000 Zagreb, Croatia; miletic@sfzg.hr; 2 Department of Materials, Faculty of Mechanical Engineering and Naval Architecture, University of Zagreb, 10000 Zagreb, Croatia; zdravko.schauperl@fsb.hr; 3 Division of Materials Chemistry, Ruđer Bošković Institute, 10000 Zagreb, Croatia; mmarcius@irb.hr

**Keywords:** remineralizing dental materials, hydroxyapatite, calcium phosphates, ion release, varnish materials, enamel remineralization, bioactive coatings

## Abstract

This study aimed to determine the effects of three different varnish materials (containing casein phosphopeptide-amorphous calcium phosphate, nano-hydroxyapatite, and fluoride) on enamel. Thirty-three extracted human third molars were used for specimen preparation. These were demineralized using phosphoric acid. Three experimental groups (n = 11) were treated with 3M™ Clinpro™ White Varnish, MI Varnish^®^, and Megasonex^®^ toothpaste, respectively, every twenty-four hours for fourteen days. Analysis of the microhardness of the specimens’ enamel surfaces was carried out via the Vickers method, and by scanning electron microscopy/energy dispersive X-ray spectroscopy (SEM/EDS). Analysis was performed at three stages: at baseline value, after demineralization, and after the period of remineralization. Data were subjected to Scheffe’s post hoc test. The mean microhardness values (HV0.1) obtained for the group of samples treated with MI Varnish^®^ were higher compared with the other two groups (*p* = 0.001 for both comparisons), while the first and third groups did not differ significantly from each other (*p* = 0.97). SEM analysis showed uneven patterns and porosities on all samples tested. EDS results showed an increase in the mineral content of the examined samples, with the highest mineral content observed in the MI Varnish^®^ group. It can be concluded that MI Varnish^®^ use has a better remineralization effect on enamel than the other two materials.

## 1. Introduction

Enamel is the outermost layer of the tooth; therefore, it is susceptible to various influences and conditions of the oral cavity. Enamel is primarily composed of hydroxyapatite (Ca_10_(P0_4_)_6_(OH)_2_) [1], which is a crystalline form of calcium phosphate. While it is the hardest tissue in the human body [2], it is highly prone to demineralization, which occurs in acidic environments and is usually accelerated by the consumption of acidic and sugar-containing foods [3]. On the other hand, antibacterial agents, saliva, and ions (e.g., fluoride, calcium, and phosphate) promote remineralization. The balance between the continual remineralization and demineralization processes is extremely fragile; thus, when disturbed for a long period, imbalance usually leads to early carious lesions [4].

The minimally invasive approach in dentistry dictates the usage of treatments that avoid the loss of hard dental tissues [5,6]. One such treatment involves the remineralization of early, noncavitated lesions, as opposed to performing conventional treatment [7]. To date, fluoride is considered the principal remedy for halting mineral loss from enamel; although, materials which can serve as reservoirs and release fluoride continuously have been developed [8,9]. The introduction of fluoride for preventative uses in dentistry was revolutionary, resulting in a substantial decline in caries prevalence [10,11]. Aside from its affinity for treatment of hard dental tissues [12], fluoride leads to the formation of fluorapatite. Fluorapatite is less susceptible to acidic effects than hydroxyapatite, as it replaces hydroxyl groups in enamel apatite [13,14]. However, the downside of fluoride usage is that it usually leads to superficial remineralization, leaving the deeper layers unaffected [15].

However, new findings on remineralization materials and mechanisms have led to the development of new courses of action concerning the promotion of demineralized enamel regeneration. The introduction of casein phosphopeptide-amorphous calcium phosphate (CPP-ACP) as a remineralizing agent marked a major breakthrough in arresting early carious lesions [16]. The usage of CPP-ACP makes the stabilization of calcium phosphate on dental surfaces and in dental plaques possible. CPP-ACP creates non-cariogenic plaques that release calcium and phosphate ions, promoting remineralization in acidic environments [17]. These findings have led to the release of varnishes containing both CPP-ACP and high amounts of fluorides.

Nano-hydroxyapatite, which resembles the structure of apatite crystals found in hard dental tissues, can replace the natural mineral content of enamel [18,19]. Its advantages are primarily due to its size, as nanosized particles dissolve better, enabling a faster reaction. Additionally, particles of that size fit better into enamel-induced defects; thus, creating a more compact surface and preventing further demineralization [20,21]. However, there is still no proof that any of the nano-hydroxyapatite-containing products are more effective than fluorides [22,23].

Enamel remineralization can be observed by testing its microhardness and conducting scanning electron microscopy in conjunction with energy dispersive X-ray spectroscopy (SEM/EDS), both of which are useful for observing the surface of enamel and determining its mineral composition [24,25].

The rising number of commercially available products for enamel remineralization can create confusion for operators, as there have not been enough studies conducted to suggest the most appropriate one. As such, this study aims to determine the effects of three widely used varnish materials which are labeled as bioactive (containing fluorides, CPP-ACP, and nano-hydroxyapatite) on enamel remineralization.

The null hypotheses are:

1. There will be no difference in the effect on enamel microhardness between the tested materials.
2. There will be no difference in the mineral composition of the specimens treated with the tested materials.
3. The micro-surface of all specimens will be the same.

## 2. Materials and Methods

The protocol for the current study was approved by the Ethics Committee of the School of Dental Medicine, University of Zagreb (No. 05-PA-30-XXVII-5/2021), Zagreb, Croatia. In total, 33 healthy human third molars were collected at the Department of Oral Surgery, School of Dental Medicine, University of Zagreb, Zagreb, Croatia.

### 2.1. Sample Preparation

Prior to use, the teeth were thoroughly cleaned using a scaler and brushes and were stored in saline solution at room temperature. The tested materials are listed in Table 1.

The teeth were embedded in autopolymerizing acrylic resin (Heraus Kuzler Gmbh, Hanau, Germany) and left to set for 24 h, forming rectangular blocks. The samples were then polished using a Mintech 233 (Presi, Le Loche, Switzerland) polishing machine at a speed of 300 RPM, with water cooling. Standard metallographic grinding paper was used, from rough to fine (P320, P600, P1200, and P2400), exposing the smooth enamel surface, in order to perform optimal microhardness testing and SEM/EDS analysis. The samples were randomly divided into three groups for the evaluation of microhardness (n = 10). One additional specimen for each group was used in order to make samples for SEM/EDS analysis. These were cut into thin slices of approximately 1.5 mm thickness using an IsoMet 1000 Precision Cutter (Buehler, Lake Bluff, IL, USA) and an IsoMet Diamond Wafering Blade with 12.7 mm arbor size and 0.5 mm thickness (Buehler, Lake Bluff, IL, USA), at a speed of 250 rounds per minute.

### 2.2. Demineralization and Remineralization Cycle

The exposed buccal enamel surface was demineralized using 37% phosphoric acid (DiaDent Group International, Chungcheongbuk-do, Korea) for three minutes. The samples were then washed and air-dried. 

Three different remineralizing agents were then applied twice a day for two minutes, using a soft applicator brush (3M ESPE, St. Paul, IL, USA), and stored in saline (Croatian Institute of Transfusion Medicine, Zagreb, Croatia) at room temperature over a period of 14 days. Saline was freshly prepared every two days.

### 2.3. Vickers Microhardness Measurement

Microhardness of samples was determined using the Vickers microhardness tester KBW 1-V (KB Prüftechnik GmbH, Hochdorf-Assenheim, Germany) and was performed at the Department of Endodontics and Restorative Dentistry, School of Dental Medicine, University of Zagreb. This method is based on observing the enamel’s resistance to plastic deformation. Microhardness was measured at three stages: baseline value, after demineralization, and after the remineralization period. The load used for the microhardness measurement was 100 g (HV0.1) and was applied for 10 seconds, as suggested by Farooq [26]. On each sample, three indents were made, and the mean value was calculated.

### 2.4. SEM/EDS Analysis

SEM/EDS analysis was performed on one specimen for each material tested at the Ruđer Bošković Institute, Division of Materials Chemistry, Zagreb, Croatia. The microscope model used was the JSM-7000F (JEOL Ltd., Tokyo, Japan), and the Inca 350 EDS System (Oxford Instruments, High Wycombe, UK) was used for EDS. Preceding the examinations, the samples were polished using a brush and air-dried. The surfaces of the samples, as well as the share of certain chemical elements, were observed.

### 2.5. Statistical Analysis

The results were analyzed using descriptive statistics (mean, standard deviation), and statistical inferences were made using a mixed-design ANOVA, which included repeated measures testing (for microhardness changes due to demineralization and remineralization) and independent sample analysis to compare differences across groups. The post hoc differences were calculated using Scheffe’s post hoc test, for analysis, where the ANOVA results were significant. Since differences between microhardness were found in different sub-samples before intervention, the effects of initial hardness were assessed using the Pearson correlation coefficient.

## 3. Results

The results of microhardness testing are shown in Table 2. The baseline values for all three groups were statistically significantly different from each other (ANOVA test for three groups regarding initial microhardness values, *p* = 0.000). All differences are statistically significant, with *p* = 0.024 for the first group vs. the second group, *p* = 0.008 for the first group vs. the third group and 0.000 s vs. third group (Scheffe’s post hoc test). The correlations between these variables are not statistically significant, which indicates that the level of hardness at the beginning does not affect the level of hardness at the end (r = −0.301, *p* = 0.106), nor does it affect the level of hardness after demineralization. This is further proven by the fact that there is no significant difference among the groups tested after demineralization (ANOVA test *p* = 0.362).

After remineralization, the differences between the groups were statistically significant (ANOVA test for three groups and the mean microhardness values after demineralization, *p* = 0.000)—in the sense that the difference was significant between the samples in the second group (MI Varnish^®^) compared with the other two (Scheffe’s post hoc test, *p* = 0.001 for both comparisons)—while the first and third groups, 3M™ Clinpro™ White Varlish and Megasonex^®^, did not differ from each other (Scheffe’s post hoc test, *p* = 0.97). 

**Figure 1:**
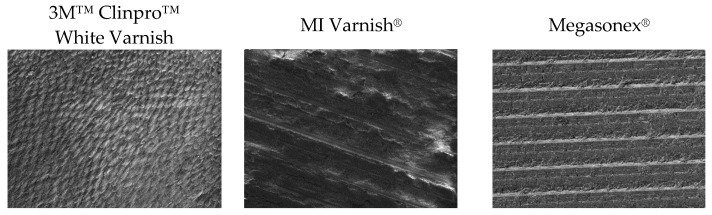
Representative SEM images (accelerating voltage of 5 kV, working distance of 10 mm, magnification of 500×) of the sample surfaces before remineralization.

**Figure 2:**
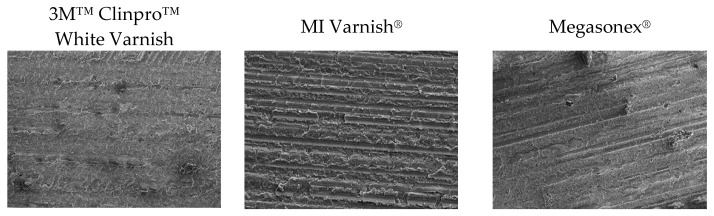
Representative SEM images (accelerating voltage of 5 kV, working distance of 10 mm, magnification of 500×) of the sample surfaces after remineralization.

SEM analysis showed normal enamel surfaces prior to demineralization (Figure 1). Much more diverse surfaces were observed after remineralization (Figure 2), with uneven patterns, porosities, material deposits, and debris.

**Figure 3:**
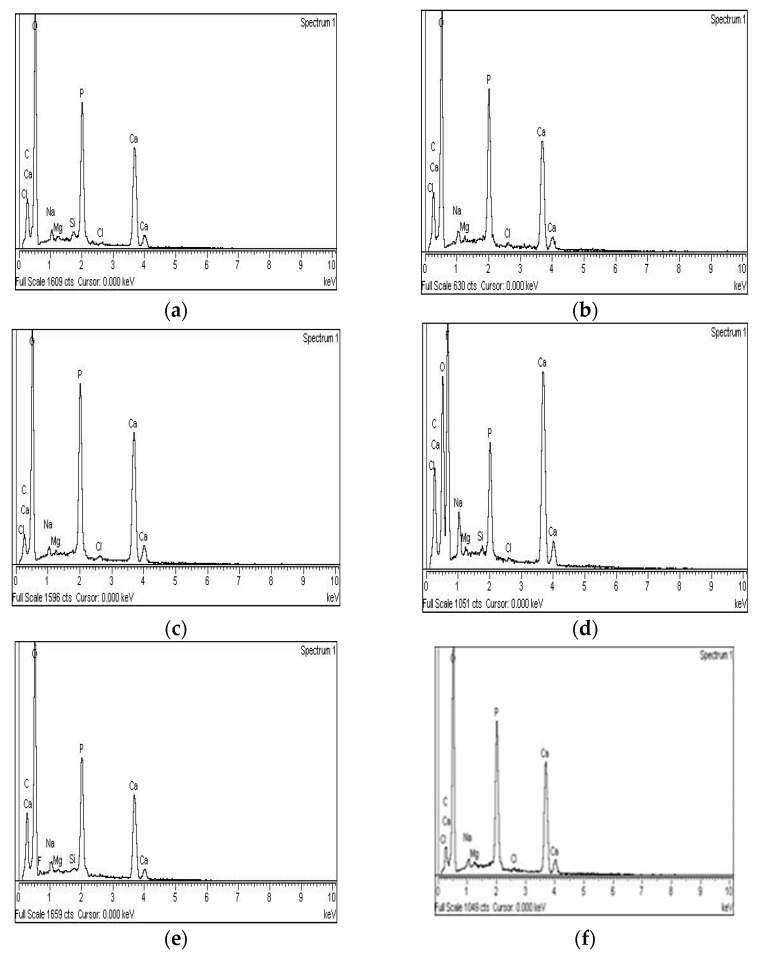
Representative results of EDS elemental analysis, following demineralization and remineralization of the samples treated with 3M™ Clinpro™ White Varnish (**a**,**b**), MI Varnish^®^ (**c**,**d**) and Megasonex^®^ (**e**,**f**).

Figure 3 shows the results of EDS analysis, before and after remineralization, with the share of particular elements varying greatly among the samples tested.

## 4. Discussion

The present study evaluated the microhardness of enamel in order to determine the extent of remineralization. This method has also been used by other authors [27,28].

In order to create an artificial demineralized lesion, 37% phosphoric acid was used, as suggested by Sorozini [29]. Within the limitations of this study, this was considered sufficient, as it has been shown that absolute simulation of oral conditions is almost impossible due to other variables, including the speed of saliva flow and its buffering ability, dynamic pH cycles in the mouth, and behavioral changes [28,30,31]. As expected, the application of acid led to a significant decrease in microhardness values due to mineral loss, lowering the values in each group to similar levels. Regarding the duration of remineralization during the trial, multiple timespans have been used: from 24 h by Ali et al. [32] to 30 days by Balakrishnan [33]. In the present study, we opted for a 14-day duration to obtain more comparable results with previous studies, as it has been shown that the extent of remineralization increases over time [33,34].

The study results show that the usage of MI Varnish^®^ leads to higher microhardness values compared with the other two materials; accordingly, the first null hypothesis was rejected.

While all three materials tested lead to certain microhardness increases, MI Varnish^®^ stands out, possibly due to its high CPP-ACP and high fluoride content. CPP-ACP binds firmly to the enamel surface and subsequently arrests fluoride ions, keeping them closer to the enamel surface for a longer period [35]. The use of fluoride as a remineralizing agent is based on its formation of fluorapatite, which requires large amounts of calcium and phosphate ions. Therefore, a lack of those ions is possible when fluoride alone is applied topically [34]. This could explain why the microhardness values obtained for 3M™ Clinpro™ White Varnish, which contains only fluorides as an active ingredient, are lower. These findings were previously confirmed by Reynolds et al. [15]. As suggested by Wegehaupt et al. [36], an increase in microhardness in both cases is caused by the accumulation of fluoride-containing compounds on enamel.

Alternatively, the results from Vyavhare et al. [37] revealed that CPP-ACP did not show superior surface remineralization compared with fluorides, claiming that it mostly causes subsurface remineralization by allowing calcium and phosphate ions to penetrate deeper; thus, leading to remineralization throughout the entire lesion [38]. However, the present study included a CPP-ACP material, which also contained fluoride ions. The combination of both CPP-ACP and fluorides creates stabilized amorphous calcium fluoride phosphate, resulting in the increased incorporation of fluoride ions and increased amounts of calcium and phosphate ions on the enamel surface [34,39,40].

When it comes to nano-hydroxyapatite-containing Megasonex^®^ toothpaste, the obtained microhardness values were similar to those of 3M™ Clinpro™ White Varnish, which contains only fluorides. Juntavee [41] and Amaechi [42] reported similar results. These findings suggest that nano-hydroxyapatite could be used as an alternative to products containing only fluoride [43]. This is important, as fluorides have recently been recognized as a possible neurotoxin [44]; therefore, increasing numbers of people are avoiding it [44]. However, further investigation of this topic is required.

Huang et al. [45] concluded that different concentrations of nano-hydroxyapatite were able to remineralize enamel at each time point in pH cycling; moreover, the optimal concentration of nano-hydroxyapatite for enamel remineralization proved to be a 10% suspension [46].

Another important factor to consider regarding the use of nano-hydroxyapatite is its particle size. A study by Huang et al. [45] showed that suspensions containing particles ranging from 10 nm to 20 nm in diameter only caused remineralization of the superficial layer in demineralized lesions. Since a previous study by Tschoppe et al. showed that nano-hydroxyapatite particles >20 nm enhance remineralization throughout the lesion [21], in the present study, the particle size was 20–50 nm in diameter.

As a method to gain insight into the changes in mineral content of the samples, SEM/EDS analysis was performed. SEM is useful for observing the changes in surface structure, whereas EDS is considered the gold standard for observing the loss and gain of minerals during the remineralization of an artificial demineralized lesion [47].

Since there were noteworthy differences in the surface microstructure and mineral content of the tested samples after remineralization, the second and third null hypotheses were partially rejected.

In order to avoid influencing the appearance of the samples’ micro-surfaces and their mineral contents, they were polished using only a brush prior to SEM/EDS. Markings made by the saw during the cutting process are visible. Additionally, clusters of different minerals and possibly additives from the tested materials can be observed. A smearlike layer, which was probably created during sample preparation, was found on all tested specimens.

EDS analysis provided insight into the chemical changes that occurred during the demineralization and remineralization processes. Following demineralization, lower concentrations of calcium and phosphate were observed, confirming the expected mineral loss. However, compared with findings by Wang et al., [48] the values in the present study are significantly higher. This is because samples in the present study were demineralized using only phosphoric acid, without pH cycling, and because the examined surface was enamel, which contains more minerals than dentine.

In all three groups, increased calcium and phosphate were observed when analyzing the EDS results after demineralization: the highest was in the MI Varnish^®^ group, where fluoride was also found. This is possibly due to the fact that CPP-ACP from the material arrested fluoride on the surface, which was then identified during analysis.

Within their limitations, such as the sample size and the lack of pH cycles, these types of in vitro studies provide insight into the effects that bioactive materials can produce, allowing an evaluation of their possible behavior in clinical settings; however, the results should be observed with a consideration of the experimental conditions. As such, further research is required to obtain more explanations and examples of their abilities.

## 5. Conclusions

MI Varnish^®^, a CPP-ACP and fluoride-based material, shows a higher capacity to remineralize enamel—as seen in its influence on the microhardness and chemical composition—compared with 3M™ Clinpro™ White Varnish and Megasonex^®^ toothpaste, which contain only fluorides and nano-hydroxyapatite, respectively, as active ingredients. The joint effect of CPP-ACP and fluorides is a promising combination and a vital agent for the treatment of demineralized lesions.

## Figures and Tables

**Table 1:** List of the materials used in the study and their respective active ingredients.

Material	Active Ingredients	Manufacturer
3M™ Clinpro™ White Varnish	22,600 ppm fluoride	3M ESPE, St. Paul, MN, USA
MI Varnish^®^	Casein phosphopeptide-amorphous calcium phosphate (CPP-ACP), 5% sodium fluoride	GC Corporation, Tokyo, Japan
Megasonex^®^	Nano-hydroxyapatite	Panaford B.V., Rotterdam, The Netherlands

**Table 2:** Microhardness of the three different groups in three stages (HV 0.1).

Microhardness Groups	Baseline	After Demineralization	After Remineralization
3M™ Clinpro™ White Varnish	366.00 ± 18.93	190.30 ± 23.71	236.57 ± 19.41
MI Varnish^®^	343.52 ± 26.66	192.73 ± 16.37	286.65 ± 34.07
Megasonex^®^	393.05 ± 16.14	201.90 ± 15.30	237.97 ± 32.52

## Data Availability

The data presented in this study are available on request from the corresponding author.
